# Advances in Magnetic UAV Sensing: A Comparative Study of the MagNimbus and MagArrow Magnetometers

**DOI:** 10.3390/s25196076

**Published:** 2025-10-02

**Authors:** Filippo Accomando, Andrea Barone, Francesco Mercogliano, Maurizio Milano, Andrea Vitale, Raffaele Castaldo, Pietro Tizzani

**Affiliations:** 1 Institute for Electromagnetic Sensing of the Environment, National Research Council of Italy (CNR IREA), Via Diocleziano, 328, 80124 Naples, Italy; accomando.f@irea.cnr.it (F.A.); francesco.mercogliano001@studenti.uniparthenope.it (F.M.); castaldo.r@irea.cnr.it (R.C.); tizzani.p@irea.cnr.it (P.T.); 2 GAIA iLAB, Piazzale E. Fermi, 1, 80055 Portici, Italy; andrea.vitale@cnr.it; 3 Department of Information and Communication Technology and Engineering, University of Naples Parthenope, Centro Direzionale Isola C4, 80143 Naples, Italy; 4 Department of Earth, Environmental and Resources Science (DiSTAR), Via Vicinale Cupa Cintia, 21, 80126 Naples, Italy; maurizio.milano@unina.it

**Keywords:** UAV magnetometry, drone geophysics, magnetic gradiometer, magnetic anomaly detection

## Abstract

The integration of miniaturized magnetometers with Unmanned Aerial Vehicles (UAVs) has revolutionized magnetic surveying, offering flexible, high-resolution, and cost-effective solutions for geophysical applications also in remote areas. This study presents a comparative analysis of two configurations using UAV-borne scalar magnetometers through several surveys conducted in the Altopiano di Verteglia (Southern Italy), chosen as a test-site since buried pipes are present. The two systems differ significantly in sensor–platform arrangement, noise sensitivity, and flight configuration. Specifically, the first employs the MagNimbus magnetometer with two sensors rigidly attached on two masts at fixed distances, respectively, above and below the UAV, enabling the vertical gradient estimation while increasing noise due to proximity to the platform. The second involves the use of the MagArrow magnetometer suspended at 3 m below the UAV through non-rigid ropes, which benefits from minimal electromagnetic interference but suffers from oscillation-related instability. The retrieved magnetic anomaly maps effectively indicate the location and orientation of buried pipes within the studied area. Our comparative analysis emphasizes the trade-offs between the two systems: the MagNimbus-based configuration offers greater stability and operational efficiency, whereas the MagArrow-based one provides cleaner signals, which deteriorate with the vertical gradient computation. This work underscores the need to optimize UAV-magnetometer configurations based on environmental, operational, and survey-specific constraints to maximize data quality in drone-borne magnetic investigations.

## 1. Introduction

Magnetic surveys are widely recognized in applied geophysics as a rapid, non-invasive, and cost-effective approach for subsurface exploration. Their applications span geological investigations, archaeological prospecting, and engineering surveys. Traditionally, until the early 2000s, researchers primarily conducted magnetic data acquisition using either manned aerial platforms (airplanes and helicopters) or terrestrial ground-based surveys. Both strategies, while effective within their specific frameworks, suffer from intrinsic limitations. Manned airborne surveys, although capable of covering vast areas efficiently, are generally constrained to flight altitudes exceeding approximately 100 m, which inevitably reduces the achievable spatial resolution [1,2,3]. In contrast, ground-based magnetic surveys ensure high-resolution datasets but are inherently labor-intensive, time-consuming, and restricted to accessible terrains.

The emergence of Unmanned Aerial Vehicles (UAVs, or drones) have profoundly transformed this paradigm. Recent technological advancements in UAV platforms and miniaturized geophysical sensors have enabled a new class of magnetic surveys that combine the advantages of aerial and terrestrial methods [4]. UAV-based surveys provide intermediate resolution and coverage capabilities, reduce operational costs and time, extend exploration to otherwise inaccessible terrains, and minimize risks for field personnel [5,6]. Consequently, UAV-borne magnetometry is gaining increasing traction within a broad spectrum of geoscientific applications, including mineral prospecting [7,8,9,10,11,12,13,14], unexploded ordnance (UXO) detection [15,16,17], engineering surveys such as oil infrastructure decommissioning and pipeline detection [18,19,20,21], geological investigations including volcanic and tectonic monitoring [22,23,24,25,26], and even archaeological prospection where anomalies are subtle and require fine spatial resolution [27,28,29,30,31].

Despite its rapid development, UAV magnetometry continues to face several challenges that compromise data quality and survey reliability. A primary source of error arises from magnetic and electromagnetic interferences generated by the UAV platform itself, including static distortions due to onboard electronics and dynamic disturbances induced by propeller rotation and drone maneuvers [32]. The most common strategy to mitigate such noise consists of physically distancing the magnetometer from the UAV body, typically by suspending the sensor 3 m below the aircraft using non-rigid ropes [18]. While this configuration effectively reduces interference, it introduces other critical issues such as deterioration in flight stability under windy conditions and oscillation-induced periodic artifacts in the recorded data. In addition to platform-related disturbances, abrupt movements and vibrations of the UAV exacerbate noise levels, especially when vector magnetometers (e.g., fluxgate or SQUID sensors) are employed. Although scalar magnetometers, such as proton precession, Overhauser, and optically pumped magnetometers, are less sensitive to directional oscillations, their recorded signals are still vulnerable to noise, particularly when computing horizontal or vertical gradients of the magnetic field. These gradients are essential for enhancing shallow-target detection but can be severely compromised by instability. Hence, the identification of a robust UAV-magnetometer configuration that balances noise suppression, gradient estimation capabilities, and operational efficiency, represents a priority task.

Although several studies have already investigated the use of UAV magnetometry, most of the contributions have either concentrated on optimizing flight trajectories while relying on a single magnetometer type or have primarily addressed the issue of post-processing filtering to reduce UAV-induced noise. Only limited efforts have been devoted to a systematic evaluation of different sensor types integrated into distinct UAV configurations, especially when the simultaneous measurement of the total magnetic field and its vertical gradient is required.

The novelty of the present study lies precisely in this comparative approach. We analyze, under identical environmental and geological conditions, two fundamentally different scalar magnetometer systems mounted on the same UAV platform. The first system, based on the MagNimbus magnetometer, employs a vertical gradiometer configuration with two rigidly attached sensors located above and below the UAV and supported by a radar altimeter for terrain-following capabilities. The second system, instead, utilizes the MagArrow magnetometer suspended 3 m below the UAV by non-rigid ropes, with flight altitude controlled through a Digital Terrain Model (DTM). By comparing these two approaches at the same test site, characterized by buried steel pipelines that serve as reliable reference targets, we aim to provide an unprecedented insight into how sensor type and mounting configuration affect both data quality and vertical gradient estimation.

This study contributes to the ongoing development of UAV-borne magnetic exploration for several reasons. First, it offers a direct comparative evaluation of two of the most widely employed UAV-magnetometer systems, thereby highlighting the practical trade-offs between platform stability, noise suppression, and operational efficiency. Second, it demonstrates how sensor placement and integration strategies affect the acquisition of both the total field measurements and vertical gradient data, which is critical in detecting the shallow targets. Third, through field-based validation in the Altopiano di Verteglia (Southern Italy), the study provides a realistic assessment of system performance under actual operating conditions, with buried pipelines serving as controlled test anomalies. Finally, the analysis clarifies how different system choices translate into advantages or limitations for specific survey objectives, thereby offering valuable guidance to researchers and practitioners when selecting UAV-magnetometer configurations. This study is not intended to be an alternative to the already performed tests reported online by the magnetometer manufacturers (e.g., https://www.sphengineering.com/news/ accessed on 17 September 2025), but it aims to enrich the manufacturers testing activities with additional configuration and complementary analyses.

After this introduction, the paper is structured to ensure a logical flow from the instrumentation employed to the results obtained and their interpretation. Section 2 introduces the technical specifications of the MagNimbus and MagArrow magnetometers, their integration with the UAV platform, and the design of the experimental survey. Section 3 presents the acquired datasets together with the processing workflow adopted to identify and mitigate the various sources of noise. Finally, Section 4 summarizes the main conclusions of the work, emphasizing the relevance of our findings for future developments and further research in UAV magnetometry.

## 2. Materials and Methods

We first describe the technical features of the used UAV-conceived scalar magnetometers: the MagNimbus and MagArrow; then, we introduce the settings of the configured UAV measurements systems; finally, we show the details of the performed surveys.

### 2.1. Magnetometers Technical Specifications

**Figure 1:**
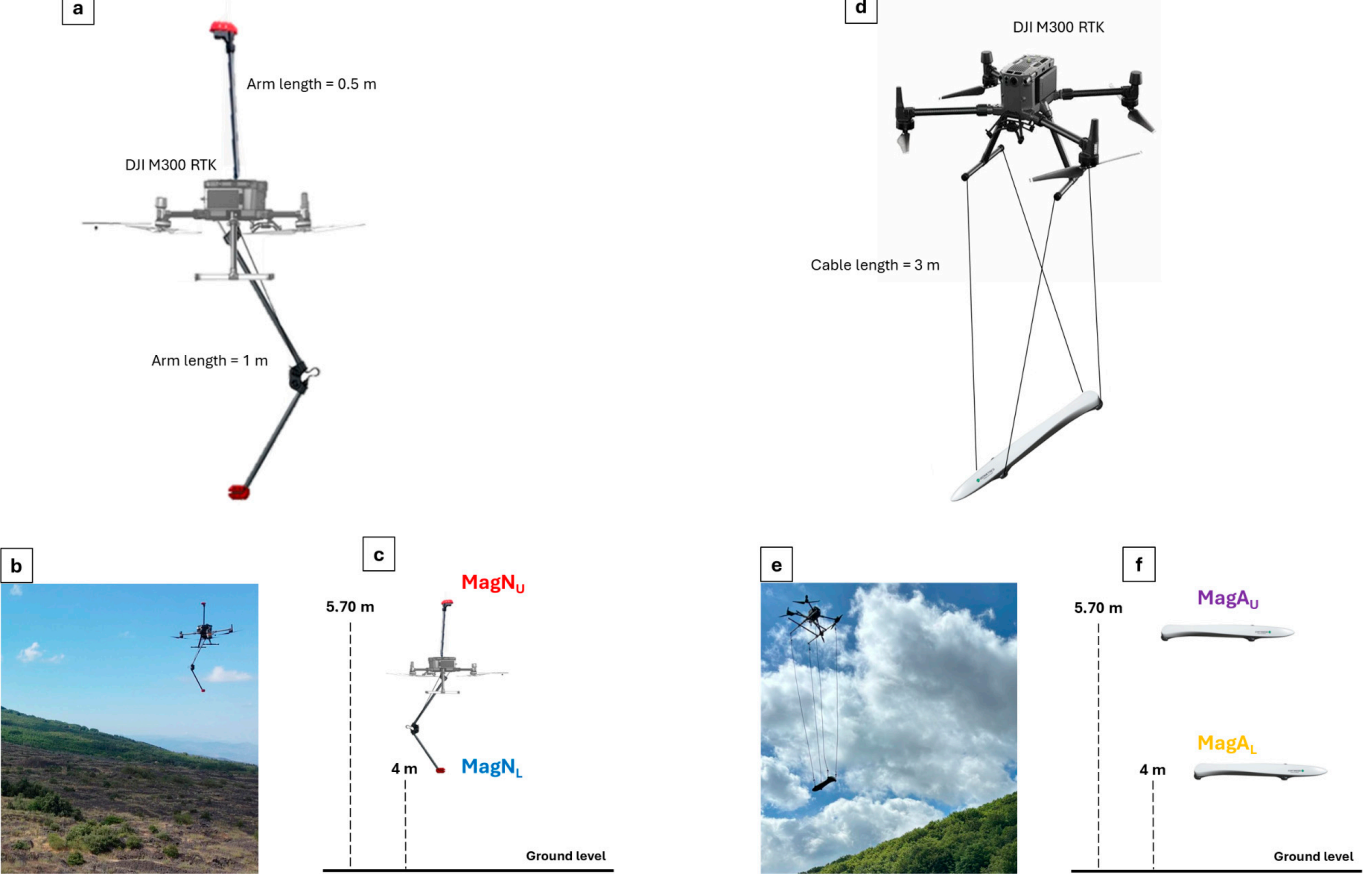
Flight configuration systems proposed for drone-borne magnetic survey. (**a**) MagNimbus-based configuration with sensors rigidly fixed to the DJI M300 RTK (manufactured by DJI Sciences and Technologies Ltd., Shenzhen, Guangdong, China); (**b**) MagNimbus-based system after the take-off and (**c**) the related survey details for the vertical gradient computation; (**d**) MagArrow-based configuration with the magnetometer suspended below a DJI M300 RTK drone; (**e**) MagArrow-based system after the take-off and (**f**) the related survey for details for the vertical gradient computation.

The MagNimbus magnetometer is produced by SPH Engineering (https://www.sphengineering.com/, headquartered in Riga, Latvia) and consists of 2 QuSpin Total-Field Magnetometer (QTFM) Gen-2 atomic sensors (manufactured by QuSpin Inc., Louisville, CO, USA) assembled in the framework of 2 split arms (Figure 1a–c). The first one is a 1 m long foldable mast, mounted below the UAV at a distance of approximately 1 m from its base. The second one is a 0.5 m long fixed mast, mounted above the UAV and distant from its top by about 0.5 m. This allows the evaluation of the vertical gradient of the total magnetic field performing one flight. The total weight is about 1 kg, and the very low sensitivity is equal to 0.003 nT. QTFM Gen-2 sensors have a high sampling rate also up to 1000 Hz. However, a 200 Hz sampling rate is recommended for the MagNimbus system, especially in the case of vertical gradient measurements. Data collection is managed with the UgCS SkyHub (manufactured by SPH Engineering, headquartered in Riga, Latvia) on-board computer, and the related software, which also allows the magnetometer to be powered by the drone’s batteries.

The MagArrow magnetometer is manufactured with the Micro-Fabricated Atomic Magnetometer (MFAM) scalar atomic sensor, produced by Geometrics (https://www.geometrics.com/, San Jose, CA, USA) (Figure 1d–f). It is characterized by compact size (1 cubic inch, 0.23 kg) and low power consumption (2.5 W). It has high sensitivity of 1pT/√Hz and high sampling frequency of 1000 Hz. The MFAM sensor has only a polar dead zone, meaning it generates a weak or no signal if aligned within ±35 degrees of the Earth’s magnetic field vector. Outside this zone, it measures the field magnitude almost independently of the orientation. The bird containing the MFAM is robust, weather-resistant and protects the sensors from environmental conditions and optimizes airflow to minimize aerodynamic interference, ensuring precise and reliable data acquisition. However, these features strictly depend on the flight configuration.

### 2.2. Settings of the Configured Systems

Both configurations are based on the use of a DJI M300 RTK drone (manufactured by DJI Sciences and Technologies Ltd., Shenzhen, Guangdong, China). The first employs the MagNimbus magnetometer as payload, whereas the second makes use of the MagArrow.

The MagNimbus-based configuration (Figure 1a–c) involves two sensors rigidly attached to the drone, which ensures greater stability and security but places them very close to the aircraft, leading to the acquisition of highly noisy signals. Considering approximately 0.2 m vertical length of the chosen UAV body, the vertical gradient measurement is managed in one flight with sensors spaced approximately 1.7 m apart. Conversely, the MagArrow-based configuration (Figure 1d–f) involves the use of the magnetometer suspended 3 m from the drone with not-rigid ropes. This helps to reduce the drone electromagnetic interference, but it also makes the system more prone to oscillation, compromising the stability of the flight system and the quality of the recorded data [24,28]. Moreover, the vertical gradient measurement is performed here by conducting two flights at different altitudes since the two sensors of the MagArrow magnetometer are placed side by side at the tail of the gray container.

While the MagNimbus-based configuration uses a radar altimeter to maintain a constant height above the ground (true terrain following), the MagArrow-based one employs a Digital Terrain Model (DTM) to ensure a constant flight height relative to the topography. This approach was chosen because the suspended use of the MagArrow magnetometer, oscillating in flight, tends to block the range of the altimeter, wherever it is installed, causing abrupt and undesirable changes in the flight path and, more critically, hazardous altitude jumps.

Finally, the sampling rate differs from one system to another. Specifically, while the MagNimbus-based configuration sampling rate is 200 Hz, the MagArrow-based one is 1000 Hz, surely allowing for high spatial resolution and direct measurement of high-frequency magnetic noise and 50 Hz fields.

The features of the configurations are summarized in Table 1.

### 2.3. Magnetic Surveys

The magnetic surveys were conducted in the Altopiano di Verteglia, a small tectonic-karst basin located in the Picentini Mountains (southern Apennines, Italy). The Terminio-Tuoro massif is characterized by a carbonate succession in platform facies, with Jurassic and Cretaceous limestones covered by Quaternary deposits, represented by discordant accumulations of pyroclastic products from the Somma-Vesuvius activity and lacustrine deposits [33,34].

The surveys were conducted with the two magnetic systems over an area of approximately 120 m × 34 m at a speed of 2 m/s. A total of 18 parallel survey lines were flown, oriented in the North–South direction with an approximate separation of 2 m among the lines. The geomagnetic field in the surveying area has a declination of 4° and an inclination of 57°. The collected data with MagNimbus-based configuration is missing two profiles due to logistical issues. Nevertheless, the quality of the work is not compromised as it aims at comparing two acquisition systems with the related pros and cons.

In order to evaluate the vertical gradient of the total magnetic field at the same flight altitude for the two considered systems, we performed two surveys with the MagArrow-based configuration at 4 (MagA_L_ in Figure 1f) and 5.7 (MagA_U_ in Figure 1f) m a.g.l., respectively, whereas we conducted a single survey for the MagNimbus-based one with the drone flying at 5 m a.g.l. and, in turn, with sensors at the same altitude of 4 (MagN_L_ in Figure 1c) and 5.7 (MagN_U_ in Figure 1c) m a.g.l.

Considering the 2 m/s drone speed and the configured sampling rate, i.e., 200 Hz and 1000 Hz for the MagNimbus- and MagArrow-based configurations, respectively, the sampling interval of the magnetic data along the survey lines was 10 mm and 2 mm for the different configurations, respectively.

## 3. Results and Discussion

**Figure 2:**
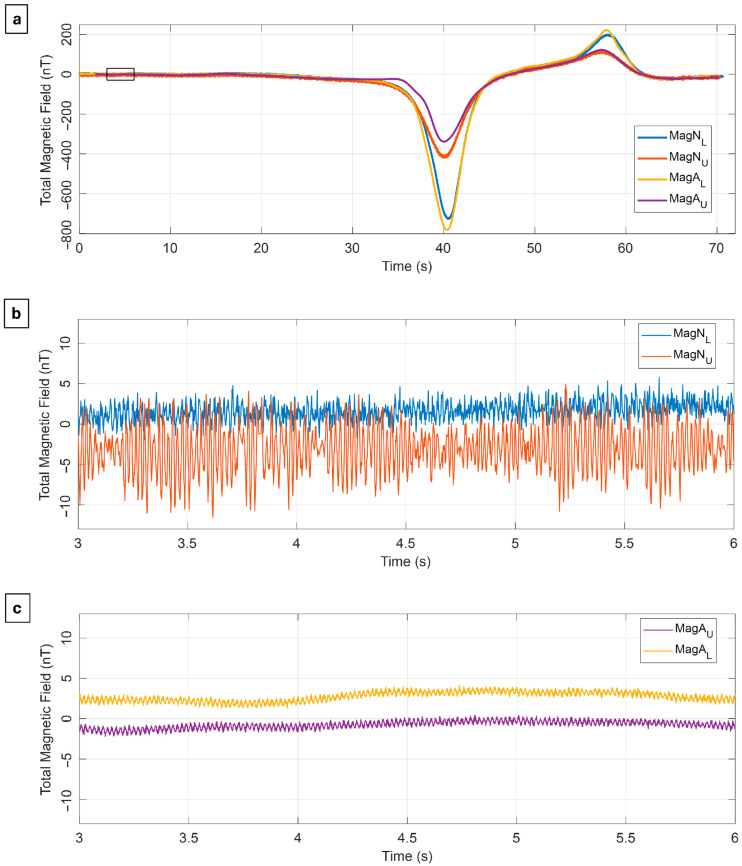
Acquired data. (**a**) Total-field anomaly data acquired along a single profile by the MagNimbus- (MagN_L_ and MagN_U_) and MagArrow-based (MagA_L_ and MagA_U_) configurations. Zoom of the acquired data using (**b**) MagNimbus- and (**c**) MagArrow-based configuration. The black box in (**a**) represents the zoomed-in region. The subscripts L and U indicate the sensors with altitude equal to 4 and 5 m a.g.l., respectively.

After applying the first step of the data processing workflow, namely the International Geomagnetic Reference Field (IGRF) correction, we show in Figure 2 the unfiltered total-field anomaly data along a single profile as function of the acquisition time, with time zero corresponding to the beginning of the profile for each dataset.

The datasets show very similar anomalies in shapes and amplitudes *(*Figure 2a). MagN_L_ (blue line in Figure 2a) and MagA_L_ (yellow line in Figure 2a) show higher-intensity anomalies with respect to MagN_U_ (orange line in Figure 2a) and MagA_U_ (magenta line in Figure 2a) due to the closer proximity to the ground and thus to the subsurface magnetized sources.

A closer inspection of the recorded signals reveals the presence of high-frequency noise in the dataset with amplitude depending on the distance between sensors and drone (Figure 2b,c). Indeed, MagN_U_ placed only 0.5 m from the drone shows oscillations with maximum amplitude of about 10 nT, while MagN_L_, placed 1 m from the drone, exhibits noise with a lower maximum amplitude of 5 nT. On the other hand, both MagA_L_ and MagA_U_, located at a distance of 3 m from the drone, show negligible noise, with maximum amplitudes below 1 nT. Therefore, the high-frequency and high-amplitude noise can be attributed to the magnetic and electromagnetic interference fields generated by the UAV platform. As demonstrated by Walter et al. [35], UAV produces sources of magnetic and electromagnetic interference signals, with the latter being proportional to the rotation frequency of the electromagnetic motor. However, at a separation distance of 3 m, the magnetic field generated by a DJI M300 RTK drone (DJI Sciences and Technologies Ltd., based in Shenzhen, Guangdong, China) appears to be sufficiently attenuated. Previous studies regarding the optimal distance between the magnetometers and UAV confirm this observation [18,35].

**Figure 3:**
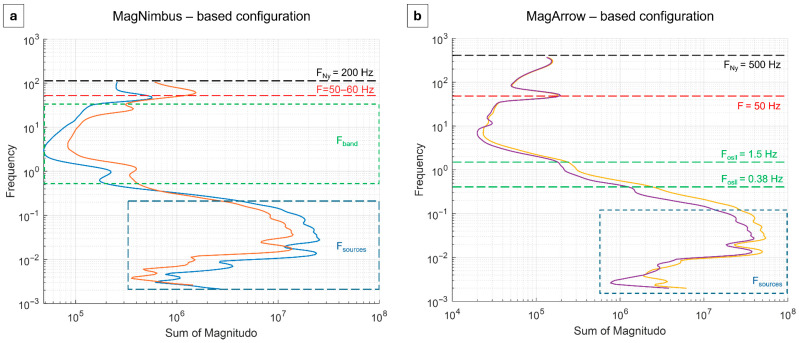
Spectral analysis. Power spectra of magnetic data from the (**a**) MagNimbus-based and (**b**) MagArrow-based UAV systems. F_Ny_ (black dashed lines), F (red dashed lines), F_band_ (green dashed box), F_osll_ (green dashed lines) and F_sources_ (blue dashed boxes) are the frequency contributions discussed in the text. MagN_L_: blue line, MagN_U_: orange line, MagA_L_: yellow line and MagA_U_: purple line.

We then analyze the spectral content of all the data acquired from both flight configurations. To do this, we employ Continuous Wavelet Transform (CWT) [36] to create a scalogram of the datasets in the time domain. This approach enables us to study the different signals that contribute to the different frequencies and to distinguish between noise and signal components. We show in Figure 3 the power spectrum for both flight configurations. In particular, the employed systems have different sampling rates; it follows that the highest frequency that can be accurately sampled without introducing errors (i.e., aliasing), namely the Nyquist frequency (defined as half of the sampling rate), is different between the two cases, i.e., 100 and 500 Hz for the MagNimbus- and MagArrow-based configurations, respectively (F_Ny_ and black dashed lines in Figure 3).

The first and most evident spectral peak is around 50 Hz (F and red dashed lines in Figure 3). In the case of the MagNimbus-based one, this frequency can be interpreted as being due to the magnetic and electromagnetic fields generated by the UAV platform. Indeed, this peak has a greater amplitude in MagN_U_, which is the sensor closest to the drone, than MagN_L_. In the case of the MagArrow-based configuration, given the 3 m distance, the 50 Hz frequency is due to the presence of alternating fields generated by the AC power lines in the area.

Additionally, at lower frequencies, the MagNimbus-based system exhibits a series of disturbance peaks in the 0.5-30 Hz range (F_band_ and green dashed region in Figure 3a) that are not well-defined or attributable to any specific source. In contrast, the power spectrum of the MagArrow-based one, for both flights, reveals two peaks at 1.5 Hz and 0.38 Hz (F_osll_ and green dashed lines in Figure 3b), which are related to the oscillation of the system suspended from the UAV, as demonstrated in [29]. Walter et al. [37] argued that the swinging of a payload suspended below a UAV can be approximated to that of an imperfect pendulum, with expected frequencies ranging between 0.4 and 0.2 Hz for cable lengths of 3–5 m. However, the suspended magnetometer does not behave as an ideal pendulum oscillating in a two-dimensional plane. External factors, such as the strong swinging induced during UAV turns at the end of survey lines or the effect of wind, may generate oscillations at different and variable frequencies. These oscillations introduce additional variations in the recorded magnetic field due to the spatial gradients of the field within the volume explored by the moving sensor. In our analysis, the contribution of these oscillations was identified as low-frequency peaks in the power spectrum of the data, which were distinguished from the spectral band associated with the target signal. Despite the small amplitude of these two peaks, thanks to the stability achieved during flight under optimal weather conditions, it is good practice to identify and eliminate them using filters.

To roughly estimate the frequency band associated with the target signal (denoted as F_sources_ and highlighted by the blue dashed region in Figure 3), we adopted the approach described in Accomando et al. [18] and Walter et al. [35]. This involves calculating the ratio between the UAV speed (2 m/s) and the horizontal extent of the anomaly, considered as half the wavelength of the signal, and multiplying it by two. Based on this method, our analysis indicates that the target signal lies within a frequency range of approximately 0.05 to 0.06 Hz. This confirms that the target signal does not overlap with the spectral content of the noise, enabling the design of a simple filter to isolate the useful signal. In our case, increasing the UAV speed to 5 m/s would have shifted the target signal frequency to around 0.125 Hz, potentially causing overlap with noise components.

This analysis highlights the critical importance of carefully selecting acquisition parameters, such as UAV speed, to ensure high-quality data collection. In this case, the choice of a lower speed was not arbitrary but based on a realistic assumption about the expected signal characteristics, and our results now provide quantitative evidence supporting that decision. Such parameter optimization is essential for maximizing signal clarity and minimizing interference, especially in drone-based geophysical surveys, where platform dynamics directly influence data fidelity.

For both cases, F_sources_ show higher intensity in MagN_L_ (blue line in Figure 3a) and MagA_L_ (yellow line in Figure 3b) measured by the sensor closest to the ground, and, therefore, to the source. This analysis allowed us to design a time-domain Hanning-window low-pass filter with a cut-off frequency of 0.1 Hz, which preserves the lowest frequency signals associated with buried sources. The separated high-frequency noise along a single representative profile is shown in Figure S1 of the Supplementary Information, confirming the maximum amplitudes <10 and <1 nT for MagNimbus and MagArrow, respectively, as above evaluated in preliminary analysis along the profile in the time domain (e.g., Figure 2b,c).

**Figure 4:**
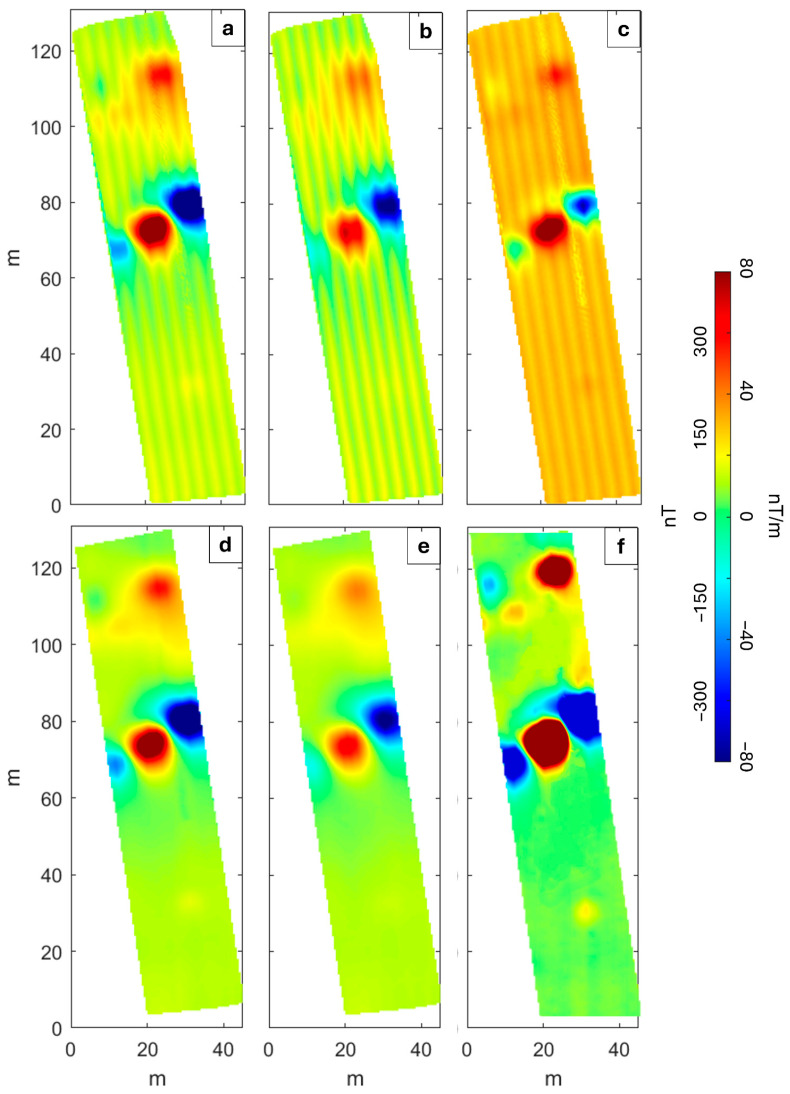
Total Field Anomaly maps from MagNimbus-based configuration. Un-filtered TFA maps retrieved from (**a**) MagN_L_, (**b**) MagN_U_ and (**c**) related vertical gradient. Filtered TFA maps at (**d**) MagN_L_, (**e**) MagN_U_ and (**f**) the related vertical gradient.

**Figure 5:**
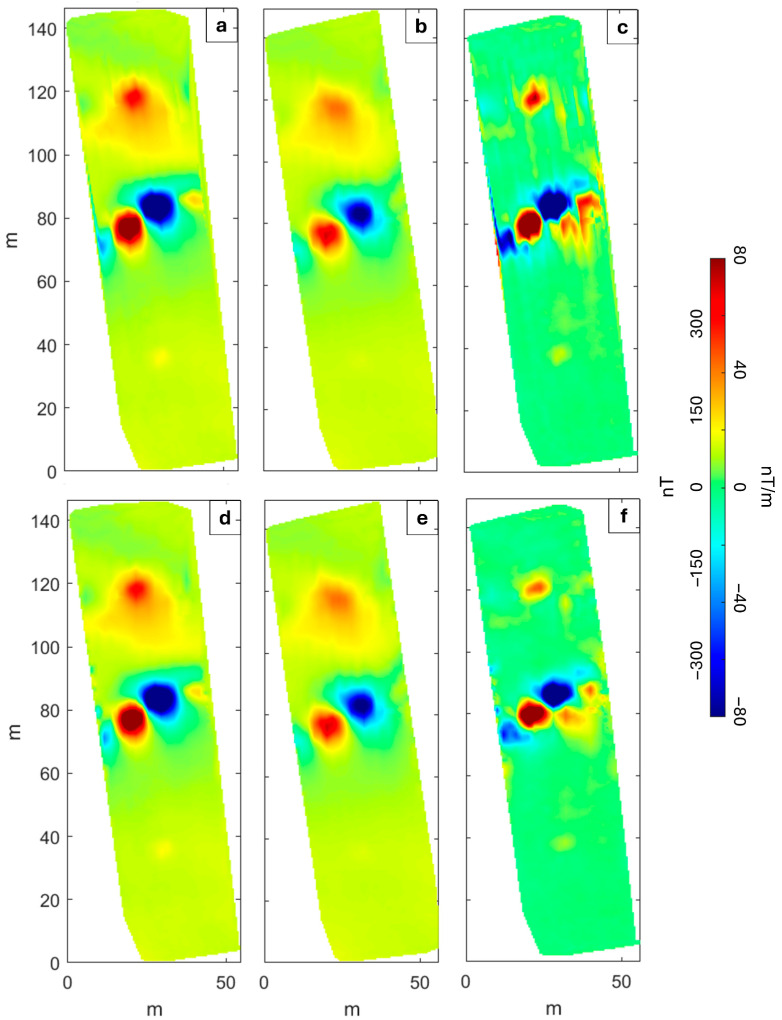
Total Field Anomaly maps from MagArrow-based configuration. Un-filtered TFA maps retrieved from (**a**) MagA_L_, (**b**) MagA_U_ and (**c**) related vertical gradient. Filtered TFA maps at (**d**) MagA_L_, (**e**) MagA_U_ and (**f**) the related vertical gradient.

We map the magnetic anomalies using a linear operator to interpolate the data recorded along the profiles considering a gridding cell of 1 × 1 m^2^. The position, amplitude, and shape of the anomalies from the raw data shown in Figure 4a–c and Figure 5a–c are similar. Specifically, small amplitude differences between the two datasets (Figure 4a–c and Figure 5a–c) can be attributed to the different noise levels, to the inaccuracy of the flight altitude and to the inaccuracy of the data position due to the different flight trajectories related to the performance of two different flights in the MagArrow-based configuration.

The main difference between the datasets collected with the two systems is that the MagNimbus-derived maps (Figure 4a–c) are characterized by a strong heading error, which depends on the variation in the orientation of the sensors during the flight and is caused by the bi-directional flight mode (South–North and North–South along adjacent lines). This error can be recognized from the different mean values of the field among the adjacent flight lines. In fact, the MagNimbus magnetometer is used in conjunction with an altimeter, which is mounted at a slight angle on the front of the drone to look forward. This setup requires the system to rotate completely between lines during data acquisition, causing a significant heading error. In contrast, the MagArrow-based configuration uses a DTM instead of an altimeter, allowing us to keep the system’s yaw fixed during flight, thereby preventing heading errors. However, we note that even the vertical gradient obtained from the unfiltered MagArrow-derived data (Figure 5c) is affected by a directional noise, which appears different from the heading error. We associate this feature with the imperfect coincidence of measurement points coordinates between the two flights.

Therefore, in both cases, after the application of the aforementioned low-pass filter, we also applied a directional filter based on the Discrete Wavelet Transform (DWT) [38,39] to the MagNimbus-derived datasets (Figure 4a–c) and to MagArrow-derived vertical gradient (Figure 5c). Although the methodological approach was the same, the degree and localization of filtering were adapted to the specific type of noise. In the case of the strong heading error, a relatively strong and uniform filtering across the entire map was required to compensate for the amplitude and systematic distribution of the error. In contrast, for the MagArrow-derived vertical gradient maps, the filtering was selective and localized only on the distorted anomalies, caused by the imperfect coincidence of measurement point coordinates between the two flights. Thus, the DWT provided a flexible approach capable of addressing both widespread noise and localized distortions, confirming the versatility of the technique as previously demonstrated in [40,41]. We represent in Figure S2 of the Supplementary Information the maps of the separated heading error for both the sensors of the MagNimbus magnetometer. This allows estimating a static offset with maximum amplitude of ±30 nT between the opposite flight lines.

The filtered Total Field Anomaly (TFA) maps (Figure 4d,e and Figure 5d,e) show a maximum amplitude variation of about 700 nT, and we identified two main groups of anomalies: the first trending NE–SW and located in the central sector that displays the greatest amplitudes; the second in the northern sector approximately trending in an E–W direction. We associate these with the presence of buried pipes. Indeed, the magnetic anomalies exhibit the typical alternating magnetic highs and lows that can be measured above a metal pipe [42]. Each section of metal pipes acquires permanent magnetization during manufacturing, forming individual magnetic dipoles. The direction of magnetization varies from one pipe section to another depending on the pipe’s orientation, which results in systematic variations in the anomaly amplitudes and/or signs above a pipeline. Finally, we observe that the filtered vertical gradient map obtained from the MagNimbus-based configuration (Figure 4f) shows better resolution compared to that from the MagArrow-based one (Figure 5f), most likely because the latter was derived from two separate flights. In fact, the filtered vertical gradient map from the MagNimbus-based system provides a clearer definition of the anomalies in the northern part of the survey area. This improved resolution suggests that there may be two distinct linear trends, rather than just one, which appear less evident in the MagArrow-derived map.

Moreover, the use of the MagArrow required two separate flights to attempt gradient calculation. This not only resulted in greater time expenditure (one flight for MagNimbus versus two for MagArrow), but it also compromised one of the fundamental principles: acquiring data simultaneously [43]. Consequently, the gradient calculation for the MagArrow is not independent of external field effects. Other strategies to compute the vertical gradient include the use of single sensor data and the well-known filter in the wavenumber domain, deriving from potential theory (e.g., [44]). This approach was not considered further, as it was deemed less efficient than the others.

The comparative assessment of the MagNimbus- and MagArrow-based configurations was carried out on a limited number of targets, specifically buried pipelines, which represent relatively strong magnetic sources. Therefore, the robustness of our conclusions is currently constrained to this type of anomaly. A more comprehensive evaluation of the two systems should involve additional tests on targets characterized by weaker magnetization, such as archaeological remains, where both the logistical strategies and the processing requirements can differ significantly. Only through such diversified testing it will be possible to fully assess the general applicability and performance of the proposed UAV-magnetometer configurations. Moreover, the present work focused on controlled survey areas of limited extent, while the scalability of the proposed systems to larger survey domains remains to be fully demonstrated. Addressing these limitations through further testing will provide a more complete picture of the trade-offs between different UAV-borne magnetometer configurations.

## 4. Conclusions

We compared two UAV-based magnetic survey systems using scalar magnetometers, MagNimbus and MagArrow, by testing them in the same field conditions over a known buried target. The analysis focused on differences in sensor configuration, flight design, and data quality, particularly regarding the total magnetic field and its vertical gradient.

Both systems proved effective for detecting buried magnetic targets and offer reliable alternatives to traditional ground or manned airborne surveys. However, each configuration presents distinct advantages and limitations. The MagNimbus-based system enables high-resolution gradient measurements in a single flight, offering greater operational efficiency and stable data acquisition, albeit with higher sensitivity to electromagnetic interference due to the proximity of the sensors to the UAV. This system, despite the presence of heading errors, provided a clearer definition of magnetic anomalies, especially in the northern part of the surveyed area, where two linear trends can be identified rather than just one. Conversely, the MagArrow-based configuration demonstrated superior noise suppression, benefiting from reduced platform interference. However, it required two separate flights to compute the vertical gradient, which increased the acquisition time and introduced potential misalignments in the gradient computation due to flight-to-flight variability. This resulted in a lower-resolution gradient map, despite the cleaner raw data.

Ultimately, this comparative analysis highlights there is no one-size-fits-all solution in UAV magnetometry. The optimal system depends on multiple factors, including target depth, required resolution, survey area constraints, and environmental conditions. Our findings underscore the importance of carefully balancing sensor–platform configuration, flight planning, and post-processing strategies to maximize data quality and ensure robust interpretation in drone-borne magnetic surveys.

Future developments of this study will focus on further testing the MagNimbus- and MagArrow-based configurations on different types of targets beyond buried pipelines, such as mineralized bodies, archaeological structures, and unexploded ordnance. This broader validation will allow us to better assess the versatility and limitations of each system in diverse geophysical contexts. In parallel, particular attention will be devoted to the processing chain: our intention is to automate and optimize the filtering of platform-related noise, refine vertical gradient estimation procedures, and explore advanced approaches such as adaptive signal processing and machine learning for anomaly detection and classification. These improvements will contribute to streamlining the workflow of our UAV-magnetometer systems, reducing operator dependency while enhancing both accuracy and robustness.

## Figures and Tables

**Table 1:** Main settings of the considered configurations based on the MagNimbus and MagArrow.

Feature	MagNimbus-Based	MagArrow-Based
**Magnetometer type**	QTFM Gen-2 dual sensor	MFAM
**Mounting configuration**	Rigidly mounted above and below the UAV	Suspended 3 m below UAV via rope
**Sensor-to-drone distance**	0.5 and 1 m	3 m
**Flight stability**	High: stable rigid structure	Reduced: subject to swinging oscillations
**Sensitivity**	0.003 nT	1 pT/√Hz
**Sampling rate**	200 Hz	1000 Hz
**Altitude control system**	Radar altimeter	DTM
**Yaw**	Variable: 180° rotation between lines	Fixed
**Vertical gradient acquisition**	Single flight	Two flights at different altitudes
**Electromagnetic interference**	Moderate to high	Very low
**Operational efficiency**	Higher: one flight for gradient analysis	Lower: two flights for gradient analysis

## Data Availability

Data are available on request by contacting the corresponding author.

## Supplementary Materials

The following supporting information can be downloaded at: https://www.mdpi.com/article/10.3390/s25196076/s1, Figure S1: Low-pass filtering: residuals analysis; Figure S2: Heading error.
