# Supplementary material for: Advances in Magnetic UAV Sensing: A Comparative Study of the MagNimbus and MagArrow Magnetometers
Source: Sensors (Basel). 2025 Oct 2;25(19):6076. doi: 10.3390/s25196076 (PMC12527078; doi:10.3390/s25196076)
Supplement: Supplementary file 1 [file sensors-25-06076-s001.zip › sensors-3867750-supplementary.pdf]

sensors

Supplementary Information for

Advances in Magnetic UAV Sensing: A Comparative Study of the MagNimbus and MagArrow Magnetometers

Filippo Accomando [1,2], Andrea Barone [1,2,*], Francesco Mercogliano [1,2,3], Maurizio Milano [4], Andrea Vitale [2], Raffaele Castaldo [1,2] and Pietro Tizzani [1,2]

1 Institute for Electromagnetic Sensing of the Environment, National Research Council of Italy (CNR IREA), Via Diocleziano, 328, 80124 Naples, Italy; accomando.f@irea.cnr.it, barone.a@irea.cnr.it, castaldo.r@irea.cnr.it, tizzani.p@irea.cnr.it.
2 GAIA iLAB, Piazzale E. Fermi, 1, 80055 Portici, Italy; andrea.vitale@cnr.it.
3 University of Naples Parthenope, Centro Direzionale Isola C4, 80143 Naples, Italy; francesco.mercogliano001@studenti.uniparthenope.it.
4 Department of Earth, Environmental and Resources Science (DiSTAR), Via Vicinale Cupa Cintia, 21, 80126 Naples, Italy; maurizio.milano@unina.it.

* Correspondence: barone.a@irea.cnr.it;

Introduction

This Supplementary Information file consists of the following two items:

- Figure S1. Low-pass filtering: residuals analysis.
- Figure S2. Heading error.

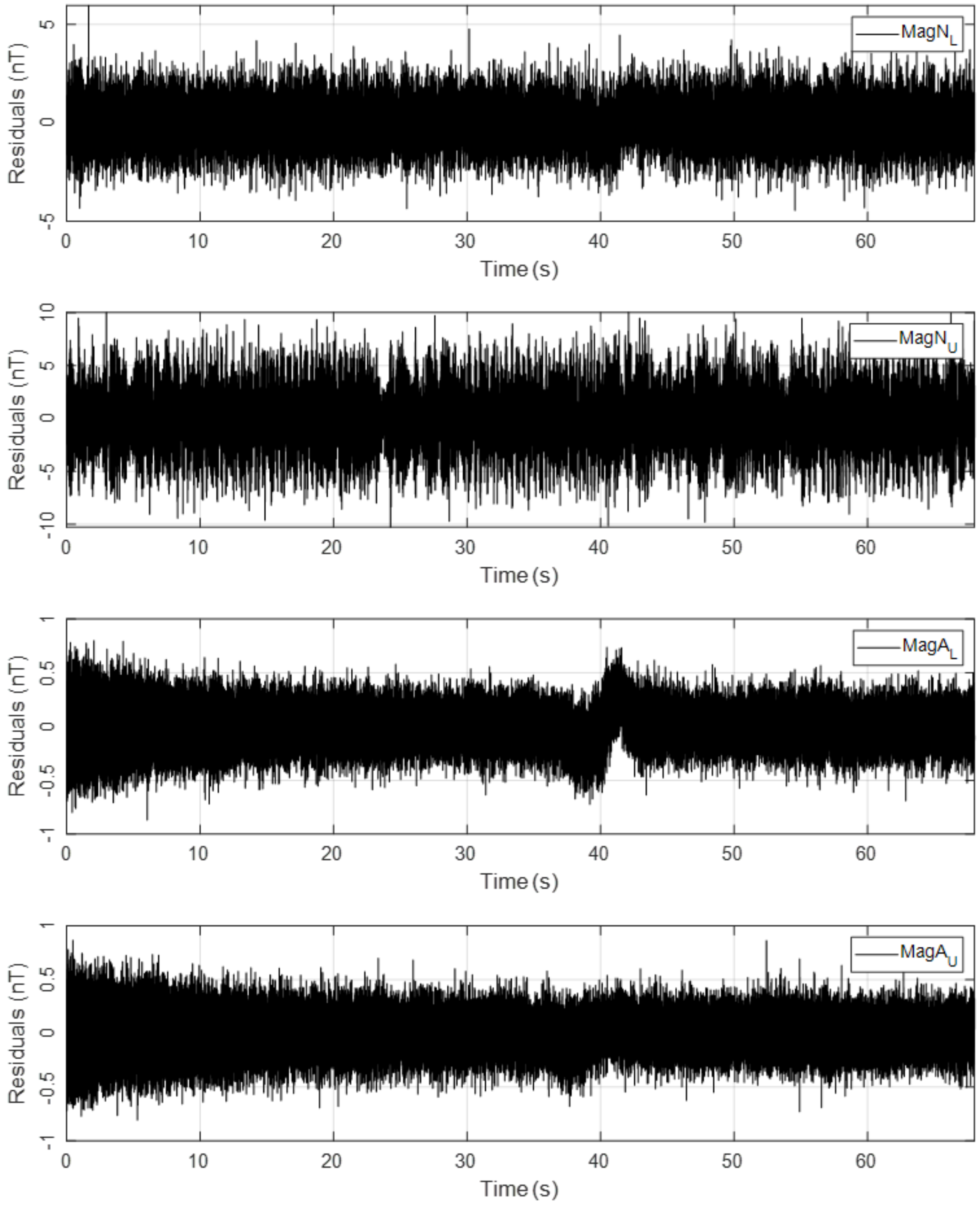

Figure S1. Low-pass filtering: residuals analysis. Difference between low-pass filtered and unfiltered signals along a single profile for the sensors (**a**) MagN$_L$, (**b**) MagN$_U$, (**c**) MagA$_L$, (**d**) MagA$_U$. Please, refer to the main text for detailed description of the performed filtering and considered sensors.

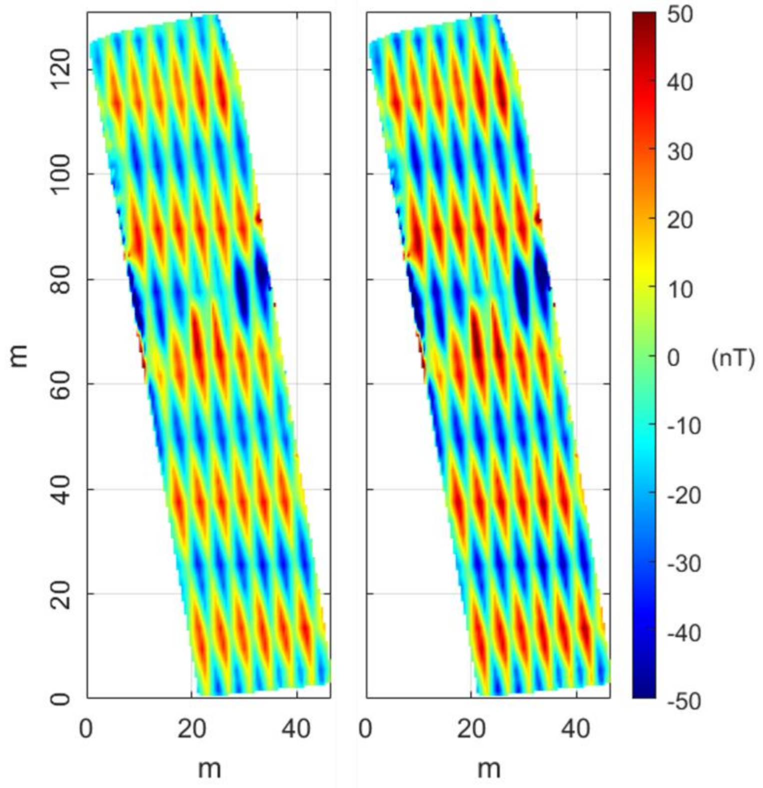

Figure S2. Heading error**.** Estimated and corrected heading error distribution from data acquired by (**a**) MagN$_L$ and (**b**) MagN$_U$ sensors. Please, refer to the main text for detailed description of the performed corrections and the considered sensors.
